# Delivery of a Small for Gestational Age Infant and Greater Maternal Risk of Ischemic Heart Disease

**DOI:** 10.1371/journal.pone.0033047

**Published:** 2012-03-14

**Authors:** Radek Bukowski, Karen E. Davis, Peter W. F. Wilson

**Affiliations:** 1 Medical Branch, Department of Obstetrics and Gynecology, University of Texas, Galveston, Texas, United States of America; 2 Agency for Healthcare Research and Quality, Rockville, Maryland, United States of America; 3 Cardiology Division, Department of Medicine, School of Medicine, Atlanta VA Medical Center, Emory Clinical Cardiovascular Research Institute, Emory University, Atlanta, Georgia, United States of America; Indiana University School of Medicine, United States of America

## Abstract

**Background:**

Delivery of a small for gestational age (SGA) infant has been associated with increased maternal risk of ischemic heart disease (IHD). It is uncertain whether giving birth to SGA infant is a specific determinant of later IHD, independent of other risk factors, or a marker of general poor health. The purpose of this study was to investigate the association between delivery of a SGA infant and maternal risk for IHD in relation to traditional IHD risk factors.

**Methods and Findings:**

Risk of maternal IHD was evaluated in a population based cross-sectional study of 6,608 women with a prior live term birth who participated in the National Health and Nutrition Examination Survey (1999–2006), a probability sample of the U.S. population. Sequence of events was determined from age at last live birth and at diagnosis of IHD. Delivery of a SGA infant is strongly associated with greater maternal risk for IHD (age adjusted OR; 95% CI: 1.8; 1.2, 2.9; p = 0.012). The association was independent of the family history of IHD, stroke, hypertension and diabetes (family history-adjusted OR; 95% CI: 1.9; 1.2, 3.0; p = 0.011) as well as other risk factors for IHD (risk factor-adjusted OR; 95% CI: 1.7; 1.1, 2.7; p = 0.025). Delivery of a SGA infant was associated with earlier onset of IHD and preceded it by a median of 30 (interquartile range: 20, 36) years.

**Conclusions:**

Giving birth to a SGA infant is strongly and independently associated with IHD and a potential risk factor that precedes IHD by decades. A pregnancy that produces a SGA infant may induce long-term cardiovascular changes that increase risk for IHD.

## Introduction

Ischemic heart disease (IHD) is an important cause of mortality and morbidity globally [1], [2]. Although methods of effective risk factor reduction are available, the prediction of IHD is limited [3]. Current risk estimation models misclassify a substantial proportion of individuals and their use in low income countries is limited [4], [5]. Moreover, those models classify 95% of women less than 70 years old as low risk, despite that almost half of them will develop IHD in their lifetime [6]. However, cardiovascular and metabolic stress associated with pregnancy provides a unique opportunity to estimate woman's lifetime risk [7]. Thus greater knowledge of IHD risk factors might improve the ability to predict vascular disease events and information related to pregnancy and childbirth may help aid prognostication for middle aged women [7].

Association between low birth weight of the offspring and increased maternal mortality and morbidity including risk of IHD has been reported [8]–[12]. However, it remains unknown if the infants low birth weight is a specific determinant of later IHD, independent of other risk factors, or a marker of general poor health. Addressing those questions is essential to evaluate the potential role of a delivery of a small infant in the risk of IHD.

The mechanism linking delivery of a low birth weight infant and greater risk of IHD in the mother is also uncertain. It has been postulated that the association between delivery of a small for gestational age (SGA) infant and the risk of IHD is due to common genetic and environmental factors, including intrauterine environment [9]–[13]. The latter was proposed because of the intergenerational correlation of low birth weight and putative intrauterine programming across generations [14]. Genetic and environmental factors tend to persist within families. Thus if SGA and IHD have common causes they would confound the relationship between SGA and IHD. Family history of IHD and related disorders would be a marker of those common causes and adjustment for family history would decrease or abolish the association between SGA and IHD.

However, pregnancy resulting in delivery of a SGA infant is associated with lower concentrations of Placental Growth Factor in maternal circulation, a factor shown to stimulate long-term angiogenesis in ischemic heart [15], [16]. Thus, it is plausible that SGA pregnancy is associated with deficiency of angiogenic placenta derived growth factors in maternal circulation, decreased angiogenesis and repair of the coronary circulation, resulting in IHD in later life. We hypothesize that delivery of a SGA infant is an independent risk factor for maternal IHD. We postulate that the association between delivery of a SGA infant and greater risk of IHD is not related to shared by both conditions genetic or environmental causes, and may result from long term cardiovascular changes initiated during a pregnancy that ended with the delivery of a SGA infant. A similar hypothesis has been proposed and long term cardiovascular changes following pregnancy have been observed in pregnancies complicated by preeclampsia [17]–[21].

## Methods

### Ethics Statement

Institutional Review Board approval was obtained and the participants gave written informed consent.

### Study design and participants

The National Health and Nutrition Examination Survey (NHANES) is a nationally representative complex-sample survey of the U.S. population, designed to provide an estimate of the health status in the general population [22]. Between 1999 and 2006 more than 40,000 individuals were surveyed using a stratified, multistage cluster-probability design. To improve reliability of the statistical estimates the survey oversampled non-Hispanic Blacks, Mexican Americans, adolescents, older adults and low-income persons. The participants underwent a household interview, physical examination and biological sample collection in mobile examination centers. The NHANES received NCHS Research Ethics Review Board Approval. A total of 6,883 female participants reported a live term birth, 275 (3.6%) of them had missing data on occurrence of IHD. Analyses were conducted in remaining 6,608 women who had delivered a term live born infant.

### Definitions

Delivery of a SGA infant was self-reported and defined as giving birth to live born infant at or after 37 weeks of gestation with a birth weight <2,500 g. The self-reported gestational age at delivery and birth weight were recorded in those prespecified categories. Age at the time of participation, self reported age at the last live birth, and at the diagnosis of first IHD event were recorded. Family history of IHD, stroke or hypertension or diabetes was reported by a participant if a diagnosis of those disorders had been made in a relative of the participant. Collected information included a history in grandparents, parents, brothers or sisters of IHD, hypertension or stroke prior to age 50, or history of diabetes at any age. Self-reported history of stroke, cancer and breast cancer were also recorded in the NHANES survey.

Risk factors for IHD were documented or measured at the time of NHANES examination. Hypertension and diabetes were diagnosed by health professionals and reported by participants. Race and ethnicity, marital status, education and income were self reported. Reported annual household income was reported in $5,000 increments. Inactivity was reported as number of hours spent daily watching television or in front of a computer. Persons who reported smoking at least 100 cigarettes in a lifetime were categorized as smokers. Current smoking status was also assessed with serum cotinine concentrations and was categorized as detectable (≥0.05 ng/mL) or non-detectable (<0.05 ng/mL). The amounts of alcohol and dietary fiber intake were determined during dietary interview recall of food consumed during the 24-hour period prior to the interview. The daily intake was estimated using the US Department of Agriculture (USDA) Food and Nutrient Databases for Dietary Studies [23]–[25]. The USDA 1994–98 Survey Nutrient Database was used to code and report the NHANES 1999–2000 data [22].

The serum concentrations of lipids and c-reactive protein were measured using methodology described previously (http://www.cdc.gov/nchs/nhanes/nhanes_questionnaires.htm). The measurements of triglycerides and low density cholesterol were made in a subsample of participants who fasted for 8 to 24 hours. The total and low density cholesterol values were categorized based on definition of dyslipidemia and high cholesterol proposed by the Adult Treatment Panel III and of high density cholesterol corresponding roughly to 10^th^ percentile observed in the NHANES [26].

### Main outcome

IHD included coronary heart disease, angina pectoris or myocardial infarction diagnosed by a physician or health professional and reported by participants. Those outcomes were previously validated among NHANES participants [5], [27], [28].

### Statistical analysis

All analyses accounted for probability sampling, stratification and clustering in the survey design. For variables that were not complete the missing values were estimated by multiple multivariate imputation. The unweighted frequencies represent prevalence of those variables in the analyzed sample. The frequencies accounting for the sampling design of the survey represent the estimated prevalence in the U.S. population. The population estimates of IHD across categories of participants' characteristics also accounted for sampling design of the survey. To compare the prevalence of IHD across participants' characteristics we used logistic regression adjusted for age and use of antihyperlipidemic medications.

Multivariable logistic regression was used to evaluate the associations between delivery of a SGA infant and the risk of IHD, cancer, breast cancer and stroke and the relationships between the risk of IHD and traditional risk factors for IHD, including family history. The adjustment for traditional risk factors of IHD was performed to demonstrate their effect on the relationship between giving birth to a SGA infant and risk of IHD. We evaluated three multivariable models of the association between SGA and IHD which adjusted for: traditional IHD risk factors, traditional risk factors and family history and risk factors used in the Framingham risk score (age, hypertension, diabetes, smoking, total and HDL cholesterol).

The association between delivery of a SGA infant and the risk of IHD was also modeled using time to event analysis. Age was used as the time scale, age at last live birth as time of study entry and age at IHD diagnosis was taken as the event. Using age at last live birth as the time of study entry allowed to account for the time interval between last live birth and diagnosis of IHD. Cumulative probability of IHD curves were constructed with the use of the Kaplan–Meier method for subjects with and without SGA infants. Hazard ratios for IHD were calculated using proportional hazard regression. Proportional hazard assumption was evaluated using global and specific tests [29].

Mortality follow-up data from the date of survey participation through December 31, 2006 were obtained from the NHANES Linked Mortality Files linking NHANES data with death certificate data found in the National Death Index.

All p values were two-sided and were considered significant if <0.05. Statistical analyses were performed with the Stata software package, version 11.1 (Stata Corporation, College Station, TX, USA).

## Results

Among 6,608 women in this study 453 had IHD. Increased risk of IHD was associated in univariate analyses with age >50 years, Black race, BMI>30 kg/m^2^, inactivity, history of smoking, detectable cotinine concentrations, low fiber diet, low educational level, lower income, presence of diabetes, elevated hemoglobin A1c, hypertension, low HDL cholesterol concentrations, high concentrations of CRP, and delivery of a SGA infant. There was a trend towards higher risk of IHD with no alcohol use compared to moderate use, single marital status and high concentrations of triglycerides. The risk of IHD was not significantly associated with total cholesterol or LDL cholesterol concentrations (Table S1).

**Figure 1:**
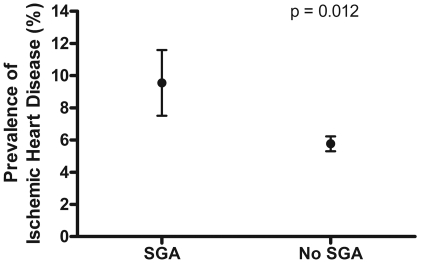
Prevalence of ischemic heart disease in relation to delivery of a small for gestational age infant. SGA, delivery of a small for gestational age infant, a term live born infant with birth weight <2500 g. No SGA, prior delivery of a term live born infant that was not small for gestational age. Population weighted proportion and 95% confidence intervals of the ischemic heart disease in women with prior live birth, adjusted for age. p-value was calculated using weighted logistic regression accounting for the sampling scheme of the survey and adjusted for age.

**Figure 2:**
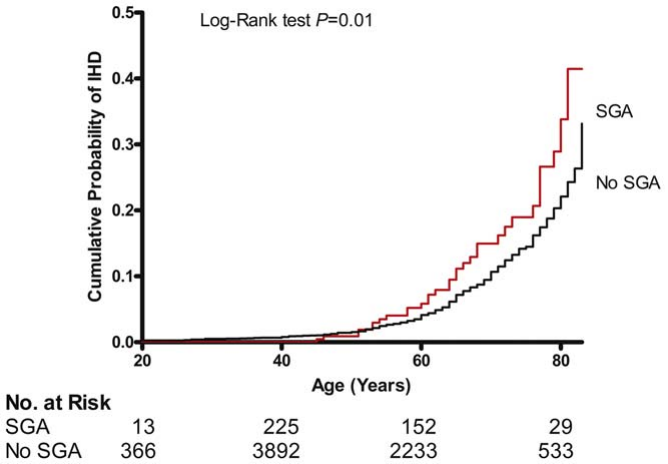
Kaplan–Meier curves of the cumulative probability of ischemic heart disease according to delivery of small for gestational age infant. SGA, delivery of a small for gestational age infant, a term live born infant with birth weight <2500 g. No SGA, prior delivery of a term live born infant that was not small for gestational age. Number at risk, number of participants at risk of ischemic heart disease (IHD) at age 20, 40, 60 and 80 years. Age was used as the time scale, age at last live birth as time of study entry and age at IHD diagnosis was taken as the event.

**Figure 3:**
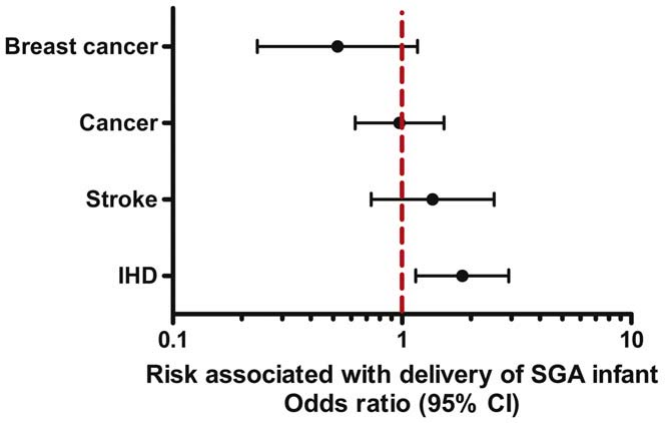
Relative odds for breast cancer, cancer, stroke and ischemic heart disease in mothers according to history of a delivery of a small for gestational age infant. Breast cancer, cancer, stroke and ischemic heart disease (IHD) were reported as diagnosed by a physician. Population weighted odds ratios and 95% confidence intervals of adverse outcomes in women with prior live term birth of a small for gestational age infant comparing to women with prior live term birth of an infant not small for gestational. The odds ratios and 95% confidence intervals were obtained using weighted logistic regression accounting for the sampling scheme of the survey and adjusted for age. SGA, delivery of a small for gestational age infant, a term live born infant with birth weight <2500 g. No SGA, prior delivery of a term live born infant that was not small for gestational age.

Three hundred nine women had delivered a SGA infant, which corresponds to the national prevalence of 4.1% (95% confidence interval, 3.5 to 4.8) among women with a prior term live birth. The relative odds for IHD was almost 2 fold higher (9.6% vs. 5.7%) in women who delivered a SGA infant (age adjusted OR; 95% CI: 1.8; 1.2, 2.9; p = 0.012) compared to women who delivered infants that were not SGA (Fig. 1). The cumulative probability of IHD among women who gave birth to a SGA infant was also significantly higher (Log-rank test p = 0.01) than among women who did not deliver a SGA infant (Fig. 2). The hazard ratio was similarly higher among women who gave birth to SGA infant than in women who did not (hazard ratio (HR), 1.6; 95% confidence interval (CI), 1.1–2.4; P = 0.019). The association was essentially unchanged among younger women, 65 years of age or younger. Delivery of a SGA infant was not associated with greater risk of cancer, breast cancer or stroke (Fig. 3).

**Figure 4:**
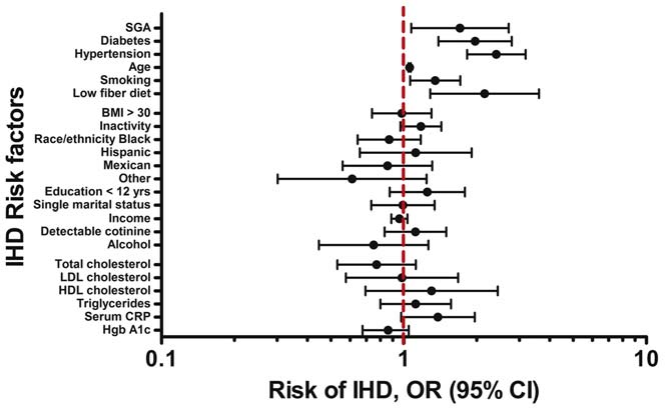
Risk of ischemic heart disease in relation to the traditional risk factors and delivery of a small for gestational age infant in a multivariable analysis. Multivariable logistic regression model of preterm birth included all investigated traditional risk factors for ischemic heart disease and SGA. IHD, ischemic heart disease. SGA, delivery of a small for gestational age infant, a term live born infant with birth weight <2500 g. No SGA, prior delivery of a term live born infant that was not small for gestational age. Smoking, smoked at least 100 cigarettes in lifetime. Low fiber diet, less than 25 g of fiber consumed daily. Antihyperlipidemic med., use of antihyperlipidemic medication at the time of examination. BMI, body mass index≥30. Inactivity, number of hours spent daily watching television or in front of the computer. Income, annual household income reported in $5,000 increments. Detectable cotinine, serum cotinine concentrations ≥0.05 ng/mL. Alcohol, no alcohol use comparing to a moderate <15 g/day alcohol consumption. Total cholesterol, total serum cholesterol ≥240 mg/dl. LDL cholesterol, LDL serum cholesterol ≥160 ng/ml. HDL cholesterol, HDL serum cholesterol <35 mg/dl. Triglycerides, serum triglycerides ≥200 mg/dl. Serum CRP, serum C-reactive protein ≥1 mg/dl. Hgb A1c, concentration of Hemoglobin A 1c >5.6%. OR (95% CI), population weighted odds ratios and 95% confidence intervals of ischemic heart disease in women with prior live term birth. The odds ratios and 95% confidence intervals were obtained using weighted logistic regression accounting for the sampling scheme of the survey and adjusted for all risk factors of ischemic heart disease.

Positive family histories of IHD, diabetes, stroke or hypertension were associated with increased risk of IHD in the NHANES women. However, the association between delivery of SGA infant and increased risk of IHD remained unaffected by adjustment for those family histories (Table 1). In multivariable analysis including all studied IHD risk factors and SGA, adjusted for use of antihyperlipidemic medications, only delivery of SGA infant, diabetes, hypertension, age, history of smoking and low fiber diet were independently associated with increased risk of IHD (Fig. 4). The risk of IHD associated with delivery of SGA infant was minimally attenuated in this multivariable model (risk factors adjusted OR; 95% CI: 1.7; 1.1, 2.7; p = 0.025) and was comparable with the strongest risk factors, hypertension and diabetes. In a fully adjusted model, accounting for all studied IHD risk factors, SGA and family history , the risk of IHD associated with delivery of a SGA infant did not change appreciably either (risk factors adjusted OR; 95% CI: 1.7; 1.1, 2.8; p = 0.022). Thus, in a multivariable model after adjustment for all studied risk factors, SGA remains independent risk factor and its effect remains essentially unchanged. In a model that included SGA and risk factors from the Framingham risk score of general cardiovascular disease the variables SGA, hypertension, diabetes, age and smoking were significantly associated with increased risk of IHD, and concentrations of total cholesterol >240 mg/dl and HDL cholesterol <35 mg/dl were not. Models with and without SGA or with and without antihyperlipidemic medications were essentially unchanged. In a fully adjusted model using continuous measurements of the covariates (age, BMI, total cholesterol, HDL, LDL, triglycerides, CRP, fiber intake) the risk of IHD associated with delivery of a SGA infant was essentially unchanged (risk factors adjusted OR; 95% CI: 1.7; 1.1, 2.9; p = 0.027).

**Table 1:** Risk of ischemic heart disease in relation to delivery of a small for gestational age infant and family history.

			Risk of IHD			
	Sample N (%)*	Population proportion (% (95% CI)†	Age adjusted OR (95% CI)‡	p	Age + FH adjusted OR (95% CI)‡	p
**SGA**	309 (4.7)	4.1 (3.5, 4.8)	1.8 (1.2, 2.9)	0.012	1.9 (1.2, 3.0)	0.011
**FH of IHD**	971 (14.7)	17.6 (16.2, 19.1)	2.5 (1.8, 3.4)	<0.0001	1.9 (1.3, 2.8)	0.001
**FH of stroke or hypertension**	1,602 (24.2)	35.1 (33.2, 37.1)	2.2 (1.7, 2.9)	<0.0001	1.8 (1.3, 2.4)	<0.0001
**FH of diabetes**	3,353 (50.7)	51.2 (49.1, 53.4)	1.7 (1.3, 2.1)	<0.0001	1.5 (1.2, 1.9)	0.003

SGA, prior delivery of a small for gestational age infant. FH, history of a first degree relative with: ischemic heart disease (IHD), stroke or hypertension, or with diabetes.

*Unweighted number and proportion of observations for each characteristic in the sample;

† Population-weighted estimates of the proportion and 95% confidence interval of the U.S. population of women with prior live birth;

‡ OR (95%CI), odds ratios, 95% confidence intervals and p-values were calculated using weighted logistic regression accounting for the sampling scheme of the survey and adjusted for age or age and family history of ischemic heart disease, stroke or hypertension or of diabetes, thus representing the risks in the U.S. population.

There was no evidence of association of SGA with traditional IHD risk factors except for smoking. The age adjusted odds of hypertension (1.3; 0.9, 1.9; p = 0.2), diabetes (0.8; 0.5–1.4; p = 0.4), elevated total and LDL cholesterol concentrations (1.1; 0.8, 1.7; p = 0.5 and 1.02; 0.6, 1.8; p = 0.9, respectively) were not significantly associated with giving birth to SGA infant. SGA was associated with significantly higher age adjusted odds of smoking (1.4; 1.04, 1.9; p = 0.028).

In a stratified analysis SGA was associated with significantly increased risk of IHD in smokers and in non-smokers. The risks of IHD among smokers with and without SGA were higher than among non-smokers. Among non-smokers the prevalence of IHD was 9.7 (5.7–16.0)% for women with SGA infant and 5.3 (4.6–6.1)% for women without SGA infant (P = 0.02). Among smokers the prevalence of IHD was 15.0 (9.5–22.9)% in women with SGA infant and 6.3 (5.1–7.6)% in women without SGA infant (P = 0.0006).

The age adjusted prevalence of IHD in women with and without SGA infant was respectively 2.4% and 1.2% among women less than 50 and 17.4% and 10.8% among women 50 years or older. The median time interval from last delivery to occurrence of IHD was 30 (interquartile range: 20, 36) years. The time interval from last delivery to occurrence of IHD was not significantly different between women with and without SGA infant (median; interquartile range: 29; 20–35 and 30; 20–39; respectively p = 0.3). Among 54 women who later died due to IHD the mean age at death was 86 for women with SGA infant, 80 for women with non-SGA infant and 79 years for all women in the NHANES population.

## Discussion

### Interpretation of the key findings

The primary finding of this study is that delivery of a SGA infant is strongly associated with increased maternal risk of IHD. In the multivariable analysis the association was independent of the family history of IHD, diabetes, stroke or hypertension as well as other risk factors for IHD. Delivery of a SGA infant preceded the presentation of IHD by a median of 30 years.

The strength of this study is its representativeness, generalizability of the findings and accounting for IHD risk factors. The sampling scheme of the NHANES has been designed to represent the entire U.S. population. Studies that previously reported an association between low birth weight and greater risk of IHD did not fully account for IHD risk factors [8]–[13]. Additionally, those studies evaluated IHD mortality as outcome, which is subject to confounding by factors that affect survival, such as socioeconomic status [8], [10]–[12], [30]. This could explain the non-specific association between delivery of a SGA infant and greater risk for all-cause mortality observed in those studies [8]–[12]. In this study SGA was significantly associated exclusively with IHD, strengthening the causal inference. However, non-significant association with stroke is likely due to smaller number of events, strokes, which occurred in the study population of relatively young women. Our analyses evaluated family history of IHD, hypertension or stroke and diabetes. Those positive family histories were strongly associated with increased risk of IHD. However, adjustment for the positive family history did not affect the relationship between delivery of a SGA infant and greater risk for IHD, suggesting that family history is not a significant confounder. Two studies reported an association between family history of premature death from IHD and delivery of a low birth weight infant and an association between increased risk of IHD in grandparents and delivery of a SGA infant, suggesting that family history could confound the relationship between delivery of a SGA infant and IHD [13], [31] However, these studies reported only a modest increase of 9% for a positive IHD history in parents and 16% increase for positive IHD history in grandparents for women who had delivered a SGA infant, indicating that the association between family history of IHD and delivery of SGA infant is relatively weak and thus unlikely to substantially confound the findings of this study [13], [31].

Traditional risk factors of IHD were evaluated in NHANES and delivery of a SGA infant was one of six risk factors independently associated with the risk of IHD in the multivariable analysis, an effect that rivaled hypertension and diabetes. The effect of delivery of a SGA infant on the risk of IHD was also independent of hemoglobin A1c level and undiagnosed diabetes [32], both potential confounders of the studied association [33], [34]. Although diabetes and elevated concentrations of hemoglobin A1c are certainly correlated they also represent different phenomena e.g. patient with elevated hemoglobin A1c might not be diagnosed with diabetes and may have either undiagnosed diabetes or glucose intolerance that do not meet the diagnostic criteria for diabetes. We found no evidence of confounding by smoking status. Although smoking is associated with giving birth to a SGA infant and with increased risk of IHD, a significant association between birth of SGA infant and increased risk of IHD was observed among smokers as well as non-smokers. Also adjustment for history of smoking as well as current cotinine concentrations in multivariable analysis did not substantially affect the association between birth of SGA infant and increased risk of IHD. The strength and independence of other risk factors, together with the ease of obtaining this information, suggest that delivery of a SGA infant could be an important determinant of later risk for IHD in women.

As a potential risk factor, delivery of SGA infant may help to predict IHD and provide newer information on the origins of atherosclerosis by identifying women at risk in their child-bearing years, preceding the occurrence of IHD by a long time [35]. Earlier identification of women at high risk for IHD would provide a longer time for risk factor reduction, which has been shown to be effective in prevention of IHD [3]. However, the findings of this study would need to be confirmed using prospective study design to allow clinical use.

It has been proposed that the association between delivery of a SGA infant and risk of IHD is due to shared genetic or environmental factors, including intrauterine environment [9], [10], [12], [13], [31]. The latter proposes that fetal programming, Barker's hypothesis, operates across generations [14]. However, two observations in this study are incongruent with the common cause hypothesis. First, genetic and environmental factors tend to persist within the family, which is known as familial aggregation [36]. A family history of IHD would be expected to be associated with both delivery of SGA infant and greater IHD risk if both resulted from common causes. Statistical adjustment for family history of IHD would be expected to attenuate the association between delivery of SGA infant and risk of IHD. We found no evidence of such attenuation. Second, shared genetic or environmental causes of SGA and IHD would imply that risk factors of IHD, including family history of IHD, would increase risk of IHD as well as delivery of a SGA infant. Adjustment for those risk factors would then attenuate or abolish the association between delivery of a SGA infant and the risk of IHD. However, such adjustment did not materially affect our findings, which would support the hypothesis that pregnancy culminating in delivery of a SGA infant may have a deleterious effect on maternal cardiovascular system and is associated with long term cardiovascular changes that culminate in greater IHD risk later in life. This reasoning is further supported by observations that preeclampsia, a placental disorder during pregnancy associated with increased risk of delivery of a SGA infant, results in impaired endothelial function and vasodilatation after the pregnancy and increased risk of developing hypertension and IHD in later life [17]–[21]. The alternative hypothesis that the SGA pregnancy could potentially be an early manifestation of cardiovascular disease is unlikely because of the observed lack of its association with the hypertension or diabetes, expressions of cardiovascular disease.

### Limitations of the analyses

The main limitation of this study is the cross-sectional design, which generally is not well suited for causative inferences because of lack of information on sequence of events and possibility of incidence-prevalence bias. However, in this study the sequence of events can be established because of known age at giving birth and age at diagnosis of IHD. Moreover, IHD is very rare in premenopausal women and thus unlikely to bias the findings due to reverse causation [37]. The incidence-prevalence bias is present if risk factor that results in death is underrepresented among those with the disease. In this study if such bias would be present women with SGA infants would be underrepresented among those with IHD, resulting in weakening of the observed association or falsely negative findings.

The retrospective assessment of birth weight could also potentially be subject to bias. However, maternal recall of infants birth weights was shown to be relatively accurate [38], [39]. The recalled birth weight was reported to be within 250 g in almost 80% of recorded birth weights and on average differ by 86 g at mean of 18 and 57 years after giving birth, respectively [38], [39]. The recalled infants' birth weights were concluded to be valuable for studies linking pregnancy history with the risk of chronic disease in later life [39]. Bias in retrospective assessment of birth weight and directionality of the association between SGA and IHD is also unlikely because of the specificity of the association. In this study giving birth to a SGA infant was associated exclusively with IHD, not with other studied disorders. The opposite would be expected if the findings would be biased.

The misclassification of exposures due to inaccuracy of self-reported preterm or term birth is also unlikely. The self-reported estimates of gestational age, decades after giving birth, were found to be sufficiently accurate for both research and clinical us. Specifically, 86% of those estimates were reported to be within 1 week and 94% within 2 weeks of gestational age recorded in medical records [40].

The pregnancy information available did not contain data on congenital malformations. However, major congenital malformations at term are uncommon and occur with a prevalence of 2–3% [41]. Thus, congenital malformations are very unlikely to result in a significantly bias and even less likely in differential misclassification.

Information on multifetal pregnancies was not available in the study. However, among the women participating in the study only 1–2% of pregnancies were multifetal [42]. Additionally, only 40% of such pregnancies deliver at term [42]. Thus, the effect of multifetal pregnancies on the findings is likely to be small.

Related to survival is possibility that higher risk of IHD in women with SGA infants is related to longer duration rather than incidence of the IHD compared to other women. This potential bias is also unlikely because women with SGA infants had mean age at diagnosis of IHD similar to women who did not deliver SGA infant (58 and 57 years, respectively). The age at death due to IHD was also similar between women who had SGA infant and in all women who died due to IHD in the NHANES population (85 and 82 years, respectively).

Some of the risk factors for IHD could have been subject to the behavioral modifications and pharmacological interventions after diagnosis of IHD. However, in this study all of the investigated risk factors were associated with the greater risk of IHD, with the exception of total cholesterol and low density lipoprotein concentrations (Table 1). Our analyses were adjusted for the use of antihyperlipidemic medications, alcohol consumption, fiber intake and amount of exercise. Information on other dietary and behavioral modifications was not available.

The associations between total cholesterol and low density lipoprotein concentrations and IHD were not statistically significant. This could reflect lower strengths of these associations observed in women [43], [44] and smaller number of participants who had those concentrations measured in this study. Alternatively, lack of a significant association could be a specific characteristic of parous women investigated in this study, because both associations were significant among all the women in NHANES study [5], [28].

In this study the delivery of a SGA infant, physician's diagnosis of IHD and family history were self reported. However, bias is unlikely because the prevalence of SGA infants in this study closely approximates the national statistics and the self reported diagnosis of IHD in the NHANES study has been previously validated [27], [45], [5]. Gestational age and birth weight were reported and analyzed using pre-specified categories rather than cut-offs selected during the analysis. The self reported family history of IHD has limited accuracy and sensitivity and specificity of approximately 75% and 90%, respectively [46]. However, the family history adds significant predictive information over traditional risk factors and in this study showed a degree of association with IHD observed in prospective studies [47].

The association with breast cancer was not significant and consistent with no effect. However, this could also be due to a small number of breast cancer events and a common causative mechanism for breast cancer and IHD acting in opposite directions. Prior studies have proposed that angiogenesis may underlie greater atherosclerosis and tumor development and could provide an alternative explanation [15]. The composite outcome of IHD, consisting of diagnosis of coronary heart disease, myocardial infarction or angina, was chosen because of a relatively small number of each individual outcome among exposed women, which would limit our ability to adjust for IHD risk factors. SGA was associated with each individual outcome coronary heart disease, myocardial infarction, angina with similar strength as with a composite outcome (age adjusted OR; 95% CI: 1.9; 1.1, 3.2; p = 0.023: 2.0; 1.002, 3.9; p = 0.049 and 1.7; 0.97, 3.0; p = 0.06).

Data on other complications of pregnancy were not available in NHANES. However, many of the common pregnancy complications are associated with fetal growth abnormalities, especially preeclampsia, pre-pregnancy chronic hypertension, and smoking in pregnancy. Furthermore, unlike other complications of pregnancy, the SGA phenotype is well defined and has better reliability and validity. Pregnancy complications also often cluster and affect the occurrence of SGA. For example, chronic hypertension increases risk of preeclampsia, and smoking decreases the risk [48], [49]. Large studies of precisely defined complications of pregnancy would be needed to determine the effect complications of pregnancy have on the risk of IHD independently of SGA.

### Implications and generalizability

In summary, delivery of a SGA infant is strongly and independently associated with later IHD in women, and potentially a risk factor that precedes the onset of IHD by decades. These results suggest that a pregnancy that produces a SGA infant induces long term cardiovascular changes that augment risk for clinical IHD. In this study we show that SGA is associated with the risk of IHD independently of traditional risk factors, but not necessarily independently of potential mediating factors and other pregnancy complications. However, birth weight is relatively easily and reliably obtainable for potential prediction of IHD in comparison to other complications of pregnancy.

Future prospective studies of IHD prediction in women accounting for their pregnancy outcomes could determine the predictive and discriminatory power of delivery of SGA infant as a risk factor in prediction of IHD.

## Supporting Information

Table S1 **Risk of Ischemic heart disease in women with prior live birth according to characteristics of participants in the NHANES 1999–2006.**
(DOC)
