# Supplementary material for: Delivery of a Small for Gestational Age Infant and Greater Maternal Risk of Ischemic Heart Disease
Source: PLoS One. 2012 Mar 14;7(3):e33047. doi: 10.1371/journal.pone.0033047 (PMC3303879; doi:10.1371/journal.pone.0033047)
Supplement: Table S1 — Risk of Ischemic heart disease in women with prior live birth according to characteristics of participants in the NHANES 1999–2006. (DOC) [file pone.0033047.s001.doc]

**Table S1. Risk of** Ischemic heart disease in women with prior live birth according to characteristics of participants in the NHANES 1999-2006.

| **Maternal characteristic** | **Sample N (%)*** | **Population proportion (%) (95% CI)†** | **Prevalence of IHD (%) (95% CI)‡** | **p-value§** |
| --- | --- | --- | --- | --- |
| **Age (y)** |  |  |  |  |
| <50 | 3,268 (49.5) ((49.5)  (9.3) | 52.6 (50.6, 54.7) | 5.3 (2.9, 7.6) |  |
| >50 | 3,340 (50.5) (14.7) | 47.4 (45.3, 49.4) | 9.3 (7.9, 10.7) | 0.022 |
| **Race and ethnicity** |  |  |  |  |
| Black | 1,278 (19.3) | 11.2 (9.3, 13.5) | 10.9 (8.4, 13.6) | 0.047 |
| Hispanic | 284 (4.3) | 5.2 (3.6, 7.4) | 10.7 (5.6, 15.8) | 0.3 |
| Mexican | 1,543 (23.4) | 7.0 (5.7, 8.7) | 8.0 (5.2, 10.9) | 0.8 |
| Other | 221 (3.3) | 4.4 (3.7, 5.3) | 6.1 (1.7, 10.6) | 0.4 |
| White | 3,282 (49.7) | 72.2 (68.8, 75.4) | 8.4 (7.1, 9.7) | referent |
| **BMI (kg/m^2^)** |  |  |  |  |
| <30 | 3,975 (60.2) | 64.4 (62.4, 66.4) | 7.6 (6.3, 8.8) |  |
| >30 | 2,494 (37.7) | 35.6 (33.6, 37.6) | 9.9 (8.1, 11.8) | 0.019 |
| missing | 139 (2.1) |  |  |  |
| **Inactivity (h/d)** |  |  |  |  |
| <1 | 986 (14.9) | 34.2 (31.7, 36.9) | 5.7 (4.0, 7.4) | referent |
| 2-3 | 1,412 (21.4) | 43.5 (41.0, 46.0) | 13.1 (10.1, 16.0) | <0.0001 |
| >4 | 810 (12.3) | 22.3 (20.2, 24.6) | 11.5 (7.7, 15.2) | 0.001 |
| missing | 3,400 (51.4) |  |  |  |
| **Smoked ever** |  |  |  |  |
| No | 4,012 (60.7) | 56.0 (54.0, 58.0) | 7.5 (6.4, 8.6) |  |
| Yes | 2,589 (39.2) | 44.0 (42.0, 46.0) | 10.1 (8.3, 12.0) | 0.003 |
| missing | 7 (0.1) |  |  |  |
| **Serum cotinine** |  |  |  |  |
| non-detectable | 3,233 (48.9) | 49.2 (46.4, 51.9) | 7.4 (6.1, 8.8) |  |
| detectable | 3,025 (45.8) | 50.8 (48.1, 53.6) | 10.2 (8.5, 11.9) | 0.006 |
| missing | 350 (5.3) |  |  |  |
| **Alcohol use** |  |  |  |  |
| None | 5,462 (82.7) | 80.6 (78.6, 82.4) | 9.1 (7.8, 10.4) | referent |
| Moderate | 465 (7.0) | 8.1 (7.0, 9.3) | 5.9 (3.2, 8.7) | 0.08 |
| Heavy | 544 (8.2) | 11.4 (10.0, 12.8) | 7.3 (4.5, 10.1) | 0.2 |
| missing | 137 (2.1) |  |  |  |
| **Fiber in diet (g/d)** |  |  |  |  |
| <25g | 5,570 (84.3) | 90.3 (89.2) | 9.2 (7.9, 10.5) |  |
| >25g | 690 (10.4) | 9.65 (8.6) | 3.5 (1.9, 5.1) | <0.0001 |
| missing | 348 (5.3) |  |  |  |
| **Marital status** |  |  |  |  |
| single | 3,584 (54.3) | 38.3 (36.6, 40.1) | 9.6 (8.1, 11.2) | 0.074 |
| married | 2,830 (42.8) | 61.7 (59.9, 63.4) | 7.8 (6.3, 9.3) |  |
| missing | 194 (2.9) |  |  |  |
| **Education (y)** |  |  |  |  |
| <12 | 2,146 (32.5) | 20.8 (18.6, 23.3) | 11.6 (9.0, 14.2) | 0.002 |
| >12 | 4,455 (67.4) | 79.2 (76.7, 81.4) | 7.6 (6.4, 8.8) |  |
| missing | 7 (0.1) |  |  |  |
| **Income tercile** |  |  |  |  |
| Low | 1,643 (24.9) | 21.1 (19.3, 22.9) | 11.3 (8.9, 13.7) | referent |
| Middle | 1,973 (29.9) | 30.7 (28.8, 32.7) | 8.0 (5.8, 10.2) | 0.04 |
| High | 2,302 (34.8) | 48.2 (45.7, 50.8) | 6.8 (5.2, 8.3) | 0.003 |
| missing | 690 (10.4) |  |  |  |
| **Diabetes** |  |  |  |  |
| no | 5,908 (89.4) | 92 (91.2, 92.7) | 7.6 (6.5, 8.7) |  |
| yes | 699 (10.6) | 8.03 (7.3, 8.83) | 14.4 (11.5, 17.3) | <0.0001 |
| missing | 1 (0.02) |  |  |  |
| **Hemoglobin A1C (%)** |  |  |  |  |
| <5.6 | 4,677 (70.8) | 77.6 (76.1, 79.1) | 5.3 (4.3, 6.3) | 0.024 |
| >5.6 | 1,716 (26.0) | 22.4 (20.9, 23.9) | 7.2 (5.9, 8.4) |  |
| missing | 215 (3.2) |  |  |  |
| **Hypertension** |  |  |  |  |
| no | 4,203 (63.6) | 66.9 (65.1, 68.6) | 5.7 (4.2, 7.1) |  |
| yes | 2,303 (34.9) | 33.1 (31.4, 34.9) | 11.0 (9.7, 12.3) | <0.0001 |
| missing | 102 (1.5) |  |  |  |
| **Total cholesterol (mg/dL)** |  |  |  |  |
| <240 | 4,966 (75.2) | 80.9 (79.5, 82.1) | 7.4 (7.5, 10.1) |  |
| >240 | 1,321 (20.0) | 19.1 (17.9, 20.5) | 8.8 (5.6, 9.2) | 0.2 |
| missing | 321 (4.8) |  |  |  |
| **Triglycerides (mg/dL)** |  |  |  |  |
| <200 | 2,493 (37.7) | 83.9 (82.5, 85.3) | 6.6 (5.2, 8.0) |  |
| >200 | 604 (9.1) | 16.1 (14.7, 17.5) | 10.0 (6.5, 13.5) | 0.067 |
| missing | 3,511 (53.1) |  |  |  |
| **LDL Cholesterol (mg/dL)** |  |  |  |  |
| <160 | 2,510 (38.0) | 86.3 (84.6, 87.8) | 6.7 (5.6, 7.9) |  |
| >160 | 420 (6.4) | 13.7 (12.2, 15.4) | 8.1 (4.5, 11.6) | 0.4 |
| missing | 3,678 (55.6) |  |  |  |
| **HDL Cholesterol (mg/dL)** |  |  |  |  |
| <35 | 256 (3.9) | 4.1 (3.6, 4.8) | 14.8 (8.2, 21.4) |  |
| >35 | 6,031 (91.2) | 95.9 (95.2, 96.5) | 8.3 (7.1, 9.4) | 0.024 |
| missing | 321 (4.9) |  |  |  |
| **CRP (mg/dL)** |  |  |  |  |
| <1 | 5,272 (79.8) | 86.1 (84.9, 87.2) | 7.9 (6.6, 9.1) |  |
| >1 | 1,048 (15.8) | 13.9 (12.8, 15.1) | 12.2 (9.6, 14.8) | 0.002 |
| missing | 288 (4.4) |  |  |  |
| **SGA** |  |  |  |  |
| no | 6,299 (95.3) | 95.9 (95.2, 96.5) | 5.7 (5.0, 6.6) |  |
| yes | 309 (4.7) | 4.1 (3.5, 4.8) | 9.6 (6.0, 13.1) | 0.012 |
| missing | 0 |  |  |  |

Subject’s age, body mass index, inactivity level, smoking history, serum cotinine concentrations, alcohol use, amount of fiber in the diet, marital status, educational level, income, diagnosis of diabetes or hypertension, hemoglobin A1C, cholesterol, triglycerides and c-reactive protein serum concentrations and history of birth of SGA child are those documented or measured at the time of examination during National Health and Nutrition Examination Survey, NHANES.

BMI, the body-mass index is the weight in kilograms divided by the square of the height in meters. Inactivity is measured in hours spent daily watching television or in front of the computer. Smoked ever, smoked at least 100 cigarettes in a lifetime. Serum cotinine was detectable if serum cotinine concentrations were ≥ 0.05 ng/mL. Alcohol use is categorized as none, moderate <15 g/ day and heavy > 15 g/day. Fiber in diet is amount of fiber consumed daily. Education is educational level in number of completed years of education categorized into < and > 12 years. Income is annual household income reported in $5,000 increments. Diabetes and hypertension are reported as diagnosed by a physician. SGA, prior delivery of a SGA infant.

*Unweighted number and proportion of observations for each characteristic in the sample; †Population-weighted estimates of the proportion of the U.S. population of women with prior live birth;

‡Population-weighted proportion and 95% confidence interval of the IHD in the U.S. population of women with prior live birth;

§ p-values were calculated using weighted logistic regression accounting for the sampling scheme of the survey and adjusted for age and use of antihyperlipidemic medications.
